# Automatic segmentation and applicator reconstruction for CT‐based brachytherapy of cervical cancer using 3D convolutional neural networks

**DOI:** 10.1002/acm2.13024

**Published:** 2020-09-29

**Authors:** Daguang Zhang, Zhiyong Yang, Shan Jiang, Zeyang Zhou, Maobin Meng, Wei Wang

**Affiliations:** ^1^ School of Mechanical Engineering Tianjin University Tianjin China; ^2^ Department of Radiation Oncology Tianjin Medical University Cancer Institute and Hospital Tianjin China

**Keywords:** automatic segmentation, brachytherapy, cervical cancer, convolutional neural networks

## Abstract

In this study, we present deep learning‐based approaches to automatic segmentation and applicator reconstruction with high accuracy and efficiency in the planning computed tomography (CT) for cervical cancer brachytherapy (BT). A novel three‐dimensional (3D) convolutional neural network (CNN) architecture was proposed and referred to as DSD‐UNET. The dataset of 91 patients received CT‐based BT of cervical cancer was used to train and test DSD‐UNET model for auto‐segmentation of high‐risk clinical target volume (HR‐CTV) and organs at risk (OARs). Automatic applicator reconstruction was achieved with DSD‐UNET‐based segmentation of applicator components followed by 3D skeletonization and polynomial curve fitting. Digitization of the channel paths for tandem and ovoid applicator in the planning CT was evaluated utilizing the data from 32 patients. Dice similarity coefficient (DSC), Jaccard Index (JI), and Hausdorff distance (HD) were used to quantitatively evaluate the accuracy. The segmentation performance of DSD‐UNET was compared with that of 3D U‐Net. Results showed that DSD‐UNET method outperformed 3D U‐Net on segmentations of all the structures. The mean DSC values of DSD‐UNET method were 86.9%, 82.9%, and 82.1% for bladder, HR‐CTV, and rectum, respectively. For the performance of automatic applicator reconstruction, outstanding segmentation accuracy was first achieved for the intrauterine and ovoid tubes (average DSC value of 92.1%, average HD value of 2.3 mm). Finally, HDs between the channel paths determined automatically and manually were 0.88 ± 0.12 mm, 0.95 ± 0.16 mm, and 0.96 ± 0.15 mm for the intrauterine, left ovoid, and right ovoid tubes, respectively. The proposed DSD‐UNET method outperformed the 3D U‐Net and could segment HR‐CTV, bladder, and rectum with relatively good accuracy. Accurate digitization of the channel paths could be achieved with the DSD‐UNET‐based method. The proposed approaches could be useful to improve the efficiency and consistency of treatment planning for cervical cancer BT.

## 1 INTRODUCTION

Brachytherapy (BT) following external beam radiation therapy (EBRT) and concurrent chemotherapy is the standard of care for patients with locally advanced cervical cancer.^1^, ^2^ Brachytherapy is an essential part of the curative intent therapy and closely associated with improvements in clinical outcomes.^3^, ^4^, ^5^ Three‐dimensional (3D) image‐based BT allows individual treatment planning based on the volumetric image of patient and is considered as a significant technical advancement and widely adopted for the treatment of cervical cancer.^6^ The application of 3D image‐based BT enables the practitioner to prescribe dose to the target volume as well as determine and potentially limit dose to the organs at risk (OARs), which is more advantageous than the conventional two‐dimensional (2D) image‐based approach. Numerous studies demonstrate improved treatment plan quality and clinical outcomes of 3D image‐based BT for cervical cancer.^7^, ^8^, ^9^, ^10^ Magnetic resonance imaging (MRI) is the preferred imaging modality for treatment planning of cervical cancer BT due to its superior soft tissue visualization relative to computed tomography (CT). However, there are many obstacles for routinely performing the MRI‐based BT in many radiation oncology departments, including limited availability, high cost, and long scanning time. Therefore, CT‐based BT of cervical cancer is widely used in treatment centers worldwide, especially in the developing countries.

Segmentation of the target volumes and OARs is an essential step in the treatment planning of 3D image‐based BT. The delineation task is usually carried out by the radiation oncologist according to the recommended guidelines. However, manual delineation of the target volumes and OARs is time‐consuming and prone to large inter‐ and intraobserver variation. Thus, accurate segmentation with high efficiency and consistency is highly desired and useful for treatment planning of 3D image‐based BT. The commonly used automatic segmentation method in clinical practice is an atlas‐based technique, which is employed by many commercial software tools.^11^, ^12^, ^13^, ^14^ To achieve automatic segmentation, atlas‐based methods rely on deformable image registration between the image to be segmented and the reference image, referred to as atlas, in which regions of interest are already segmented. The segmentation of corresponding structures in a new test image is obtained by finding the optimal transformation between the atlas and test image.^15^, ^16^ Therefore, the segmentation result strongly depends on the applied registration algorithm. The choice of a suitable and robust algorithm has a substantial effect on the result, especially in the presence of image noise and interference arising from contrast changes.^17^, ^18^ Apart from registration algorithm, the atlas itself plays a crucial role in atlas‐based segmentation. When performing atlas‐based automatic segmentation using a single atlas, accurate segmentation result cannot be guaranteed if the selected reference image is not representative or the morphology of anatomical structures is not similar enough between the atlas and test image.^19^ To reduce the uncertainties of single atlas segmentation, multiple atlases are selected from a database to register the test image, and the final segmentation result based on the multiple registrations is obtained with voting schemes.^20^, ^21^ Although multiatlas automatic segmentations improve the robustness of the segmentation results as compared to single atlas‐based ones, they are prone to topological errors and require more computational time.^16^, ^22^

Applicator reconstruction is the process of localizing the radiation source paths defined by the applicator channels in the planning images. It is another critical step during the procedure of BT treatment planning. The potential dwell positions are placed on the digitized applicator channels and corresponding dwell times are determined to meet the dosimetric objectives. Applicator reconstruction accuracy has a significant impact on the dosimetric result of the treatment plan due to the steep dose gradients of BT treatment. A small uncertainty in the digitization of applicator channels would translate into a relatively large dosimetric uncertainty.^23^, ^24^, ^25^ In general, applicator reconstruction is performed manually by the medical physicist. The digitization process is subjective and time‐consuming. Thus there is a strong need to achieve fully automatic applicator reconstruction in 3D image‐based BT to ensure treatment planning accuracy and efficiency. The applicator library integrated in the treatment planning system is the clinically available tool for automatic applicator digitization, which can significantly reduce the reconstruction uncertainty and improve efficiency. It allows channel digitization based on the manual registration of virtual applicator model with predefined source paths to its appearance in the planning images. However, the applicator library‐based reconstruction method is not fully automatic due to the manual alignment of applicator model. Moreover, applications of this method are limited to only those applicators included in the library. Electromagnetic tracking technique has been recently utilized for catheter digitization in BT.^26^, ^27^ Although this method has highly accurate digitization result, additional hardware and complex procedure may hamper its widespread application.

In recent years, convolutional neural networks (CNNs) as a kind of deep learning algorithm have been successfully applied to automatic segmentation in medical images.^28^, ^29^, ^30^, ^31^ Outstanding segmentation performance has been achieved with various architectures of CNNs. Automatic segmentation method based on CNNs is also introduced to radiotherapy treatment planning.^32^ Automatic delineation of target volumes and OARs in EBRT treatment planning for head and neck,^33^, ^34^ breast,^35^ and rectum^36^ cancer have been reported. To the best of our knowledge, there are no reports on automatic segmentation for cervical cancer BT with any CNNs. In addition, most of the CNNs utilized in the previous studies take 2D CT/MRI slices as input, thus the 3D spatial and contextual information of the whole volume cannot be utilized effectively by the networks.

In this work, we propose a novel 3D CNN architecture that is based on the popular 3D U‐Net architecture^30^ with incorporation of residual connection, dilated convolution and deep supervision (henceforth referred to as DSD‐UNET). The proposed network is trained and evaluated for automatic segmentation of high‐risk clinical target volume (HR‐CTV) and OARs in the planning CT of cervical cancer BT. Performance of the DSD‐UNET is then compared with that of the conventional 3D U‐Net. Moreover we present an automatic applicator reconstruction method based on the DSD‐UNET. The reconstruction process consists of two steps. First, the DSD‐UNET is exploited to segment all the parts of the applicator in the planning CT images. Second, segmentation of the applicator tubes is postprocessed with skeletonization and polynomial curve fitting to obtain the applicator channel paths. The feasibility and accuracy of the proposed approach are evaluated.

## 2 MATERIALS AND METHODS

### 2.A Data acquisition

A total of 91 patients with cervical cancer who underwent CT‐based BT were included in this study. All the enrolled patients received intracavitary high‐dose‐rate BT using Fletcher Williamson tandem and ovoid applicator (Elekta AB, Stockholm, Sweden). Planning CT data were acquired on Brilliance CT Big Bore (Philips Healthcare, Best, the Netherlands) system set on helical scan mode. CT images were reconstructed using a matrix size of 512 × 512 and thickness of 2 mm. The average in‐plane resolution of the CT slices is 1.11 mm (min–max range 0.97 to 1.22 mm). Only the planning CT volumes of involved patients for the first BT treatment fraction were collected for this study. The dataset for automatic segmentation study consisted of 91 CT volumes. Radiation oncologists contoured the HR‐CTV and OARs including small intestine, sigmoid, rectum, and bladder based on recommended guidelines for CT‐based BT of cervical cancer.^37^ Thirty‐two CT volumes randomly selected from the dataset for auto‐segmentation study were used to evaluate the automatic applicator reconstruction method. All parts of the tandem and ovoid applicator were carefully segmented in the planning CT images by the experienced medical physicists, including tandem tube, left ovoid tube, right ovoid tube, left ovoid, right ovoid, and cervical stopper. These manual segmentations were defined as ground‐truth (GT) segmentations and the voxels that belonged to the GT segmentations were marked and labeled.

### 2.B Preprocessing of images

**Fig. 1:**
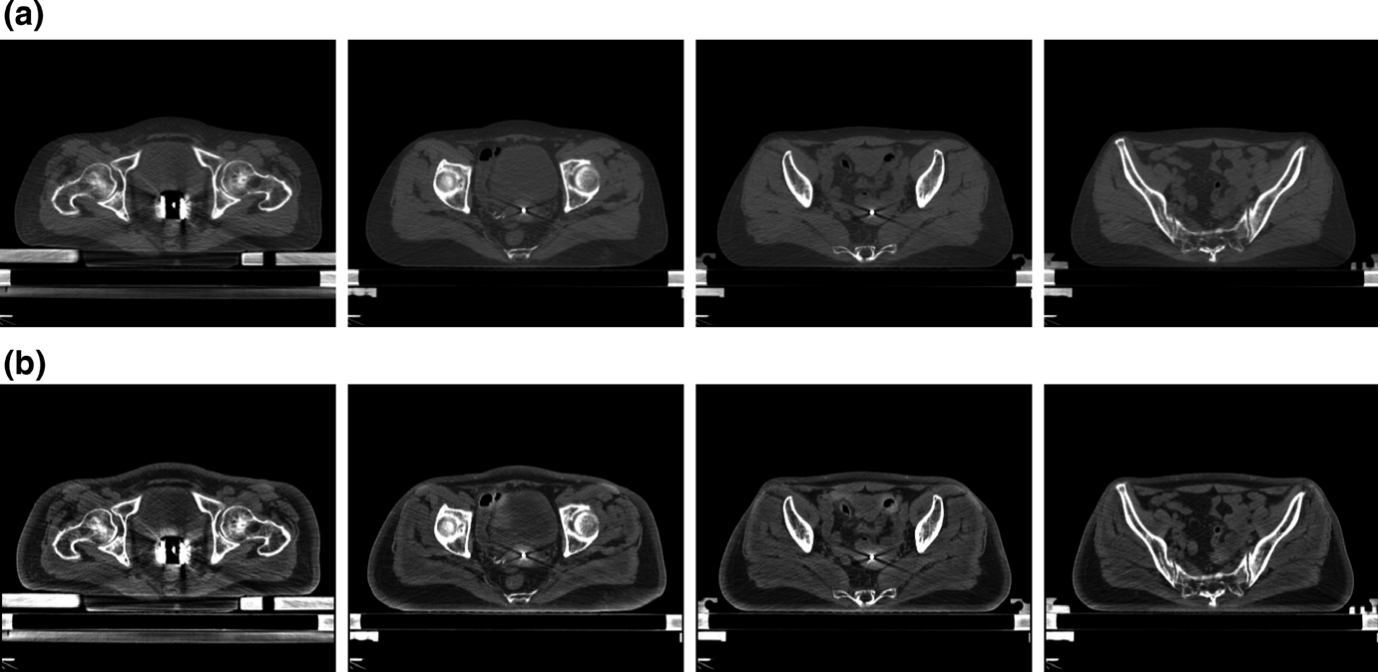
Computed tomography (CT) image enhancement using CLAHE algorithm. (a) Shows the original CT images. (b) Shows the enhanced CT images

For the segmentation of HR‐CTV and OARs, the original planning CT volumes were first cropped in each dimension to discard the regions depicting empty space or without labeled structures. Then the cropped CT volumes were resized with a linear interpolation to an identical size of 128 × 128 × 64 voxels. With the cropping and interpolating processes, the average in‐plane resolution of the obtained CT volumes is 2.89 × 2.78 mm^2^, the average interslice resolution is 2.06 mm. To enhance the image contrast of the planning CT volumes, contrast limited adaptive histogram equalization (CLAHE) algorithm was employed to preprocess the images fed to the CNN. With the CLAHE method, the shape, texture, and boundary of anatomical structures in the CT images became more distinguishable, the image quality was highly improved. Figure 1 shows the image enhancement using CLAHE algorithm. In order to preserve the original information, we kept the original CT images with the processed images using CLAHE method to compose a dual‐channel input to the proposed DSD‐UNET.

As the first step of proposed method for automatic applicator reconstruction, all parts of the applicator were segmented in planning CT images utilizing the DSD‐UNET. Due to the relatively small size of the applicator components, a fixed‐size volume of interest (128 × 128 × 80 voxels) which centered the segmented applicator was first cropped from the original planning CT volume. Then linear interpolation along the superior–inferior direction was applied to obtain a 128 × 128 × 64 CT volume with the interslice resolution of 2.5 mm. The in‐plane resolution of the resulting CT volume is identical with that of the original CT volume.

### 2.C Architecture of DSD‐UNET

**Fig. 2:**
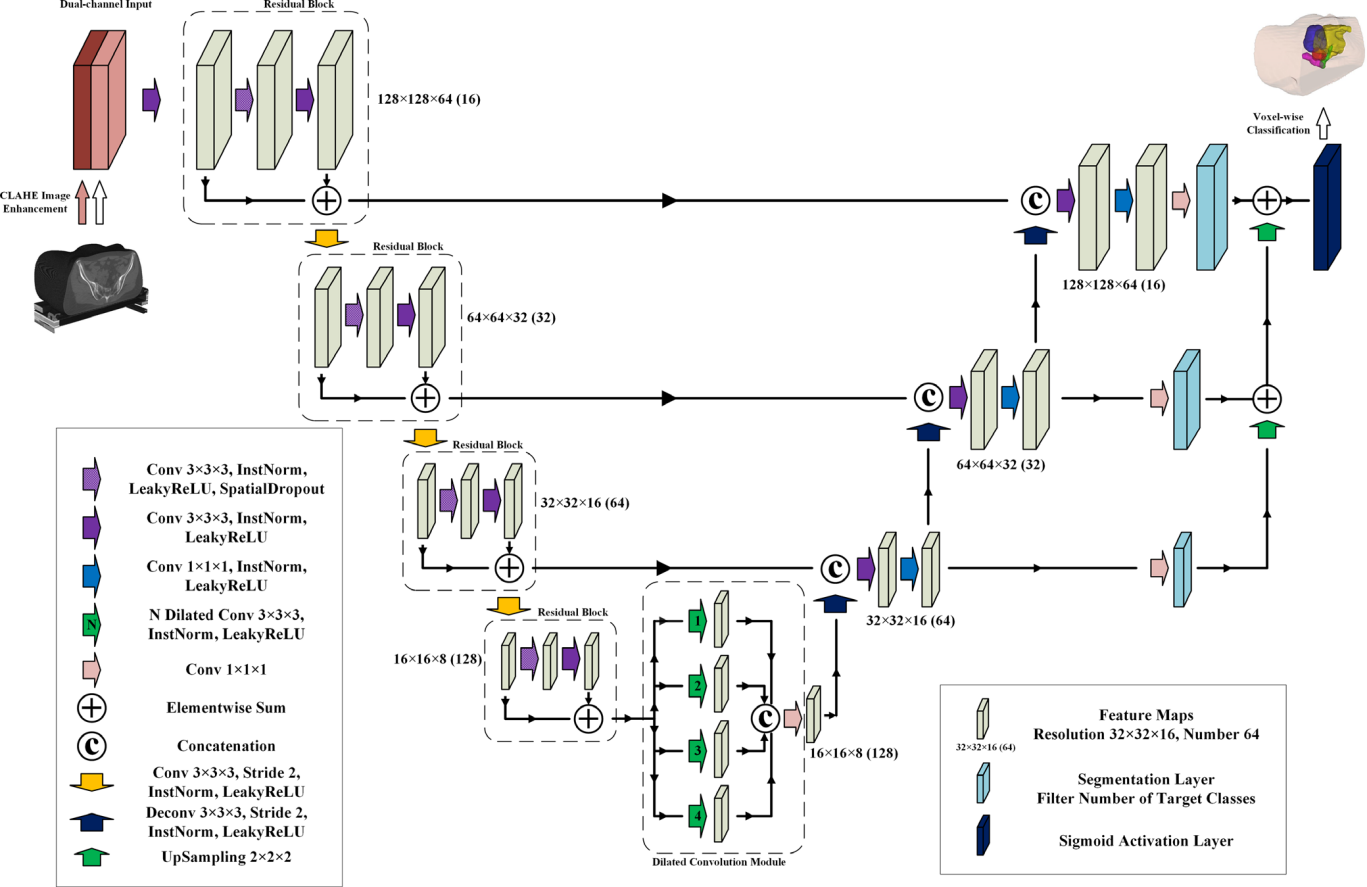
Schematic representation of the proposed DSD‐UNET architecture

The proposed novel DSD‐UNET architecture was inspired by the popular 3D U‐Net architecture. Figure 2 illustrates the detailed architecture of DSD‐UNET. Like the U‐Net, our network consisted of a contracting path and an expanding path with different stages that operate at different spatial resolutions. In order to solve the problem of vanishing gradients and accelerate the learning convergence, the residual block which consisted of two 3 × 3 × 3 convolutional layers and a spatial dropout layer in between was applied at each stage in the contracting path. The residual block was followed by a 3 × 3 × 3 convolution with a stride of 2 for downsampling. Each stage in the expanding path consisted of a 3 × 3 × 3 deconvolution with a stride of 2 for upsampling, followed by a concatenation with the feature maps from the corresponding stage in the contracting path, and then two convolutional layers with kernel sizes of 3 × 3 × 3 and 1 × 1 × 1 respectively. The segmentation layer which was a 1 × 1 × 1 convolution layer with filters equal to the segmentation classes was employed at the end of each stage in the expanding path. The number of feature channels was doubled at each stage in the contracting path and was halved at each stage in the expanding path.

Moreover, we deployed a dilated convolution module between the contracting and expanding paths, which parallel employed four dilated convolution layers with dilated factors of 1, 2, 3, and 4, respectively. With implementation of the dilated convolution module, multiscale high‐level features could be learned and aggregated to achieve more accurate and robust segmentation. Deep supervision was also employed in our network by integrating the segmentation layers at different stages of the expanding path and combining them via elementwise summation to form the final output. At last we applied sigmoid activation to this final output layer to obtain the voxel‐wise probabilities for each segmentation class. Instance normalizations^38^ followed with LeakyReLU nonlinearities were applied to all the convolutional layers through the network, except for the segmentation layers.

### 2.D Model training

The proposed DSD‐UNET architecture was implemented in Keras framework with Tensorflow as the backend. Training was performed using the Adam^39^ optimizer with an initial learning rate of 0.0005 and a decay rate of 0.5 once the learning stagnated for more than 20 epochs. The proposed network architecture was trained with batch size of 2 and maximum 200 epochs. The model with the highest performance was selected. Dice similarity coefficient (DSC) was employed as an accuracy measure of the segmentation. So we formulated a multiclass dice loss function for the model training:

(1) $$Loss_{D}=‐\frac{2}{N_{K}}\sum_{k\in K}^{}\frac{\sum_{i\in I}^{}u_{i}^{k}v_{i}^{k}}{\sum_{i\in I}^{}u_{i}^{k}+\sum_{i\in I}^{}v_{i}^{k}}$$

where $u_{i}^{k}$ represents the sigmoid activation value of the voxel i from the output filter for segmentation class k, $v_{i}^{k}$ represents the corresponding one hot encoding of the GT segmentation map. I indicates the set of voxels in the output filter and K indicates the set of segmentation classes. $N_{K}$ is the total number of segmentation classes.

In order to prevent overfitting when training the networks with limited data, data augmentation techniques including random rotations (≤10º), random scaling (≤15%), and mirroring (along left–right direction only) were applied on the fly during the training process. Due to the unavailability of sufficient GPU resource, all computations in this study were undertaken on a workstation with an Intel Xeon E5‐2620 v3 CPU (2.4 GHz, 6 cores) and 32 GB RAM.

### 2.E Evaluation metrics

When the training process was finished, performance of the model was assessed with the testing data only. The DSC, Jaccard Index (JI), and Hausdorff distance^40^ (HD) were used as the evaluation metrics of segmentation accuracy in this study. The DSC is defined as

(2) $$DSC\left(P,T\right)=\frac{2\times P\cap T}{P+T}$$

and the JI is defined as

(3) $$JI\left(P,T\right)=\frac{P\cap T}{P\cup T}$$

where P and T correspond to the predicted segmentation of the model and the GT segmentation, respectively, P∩T and P∪T represent the intersection and union of predicted segmentation and GT segmentation, respectively. HD is computed as

(4) $$HD\left(A,B\right)=\max\left(D\left(A,B\right),D\left(B,A\right)\right)$$

with D defined as

(5) $$D\left(A,B\right)=max_{a\in A}min_{b\in B}d\left(a,b\right)$$

where $d\left(a,b\right)$ is the Euclidean distance between two points, A and B indicate the measured point sets, which are defined as the voxel sets for evaluation of segmentation result. The HD measures the largest degree of mismatch between two voxel sets in the volumetric image. Thus spatial discrepancy between the automatic segmentation and GT segmentation can be quantified with this metric. In addition, the HD was used to evaluate the proposed approach of automatic applicator reconstruction. It was calculated with the points in the channel paths that determined by the developed method and manual operation.

### 2.F Experiments

The proposed DSD‐UNET architecture was exploited to complete two segmentation tasks. The first one was automatic segmentation of the HR‐CTV and OARs, another one was automatic segmentation of all parts of the applicator to finally achieve the applicator reconstruction with postprocessing. Models for the different segmentation tasks were trained and tested separately. For the segmentation of anatomical structures, the dataset was composed of 91 patient cases. It was randomly divided into a subset of 73 cases (80% of the data) for training and a subset of 18 cases (20% of the data) for testing. The evaluation metrics including DSC, JI, and HD were calculated on the testing subset. In addition, the performance of our approach was compared with that of the 3D U‐Net which was considered as the baseline for segmentation tasks in medical imaging. To make this comparison fair, the training and testing sets for 3D U‐Net model were the same as those of DSD‐UNET model. For the segmentation of applicator components, the dataset consisted of 32 patient cases. Twenty‐four cases chosen randomly were used for model training and the remaining eight cases composed the testing set. DSC, JI, and HD were computed on the testing subset to quantify the segmentation accuracy of the trained model.

Following the automatic segmentation of applicator components utilizing the DSD‐UNET, segmented applicator tubes were first postprocessed by a 3D parallel thinning algorithm^41^ to extract the central axes of these objects. This thinning algorithm proceeds by iteratively sweeping over the volumetric image, and removing voxels on object borders at each iteration until the volumetric image stops changing. An octree data structure of 3 × 3 × 3 lattice points is used to check the local connectivity and preserve the topology. When the skeletonizing process was finished, the voxel set that represented the channel axis was obtained for each of the applicator tubes. Then we fitted a 3D parametric polynomial curve of five degrees to each of these voxel sets. With the skeletonizing and curve fitting processes, index and digitization of the channel paths for the applicator were achieved. The GT channel paths of the applicator were the polynomial curves that had the best fit to the points manually determined along the applicator channels by the experienced medical physicists. To assess the accuracy of the proposed method, we used HD to measure the agreement between channel paths determined automatically and manually.

## 3 RESULTS

### 3.A Performance of the automatic segmentation of HR‐CTV and OARs using DSD‐UNET model

**Fig. 3:**
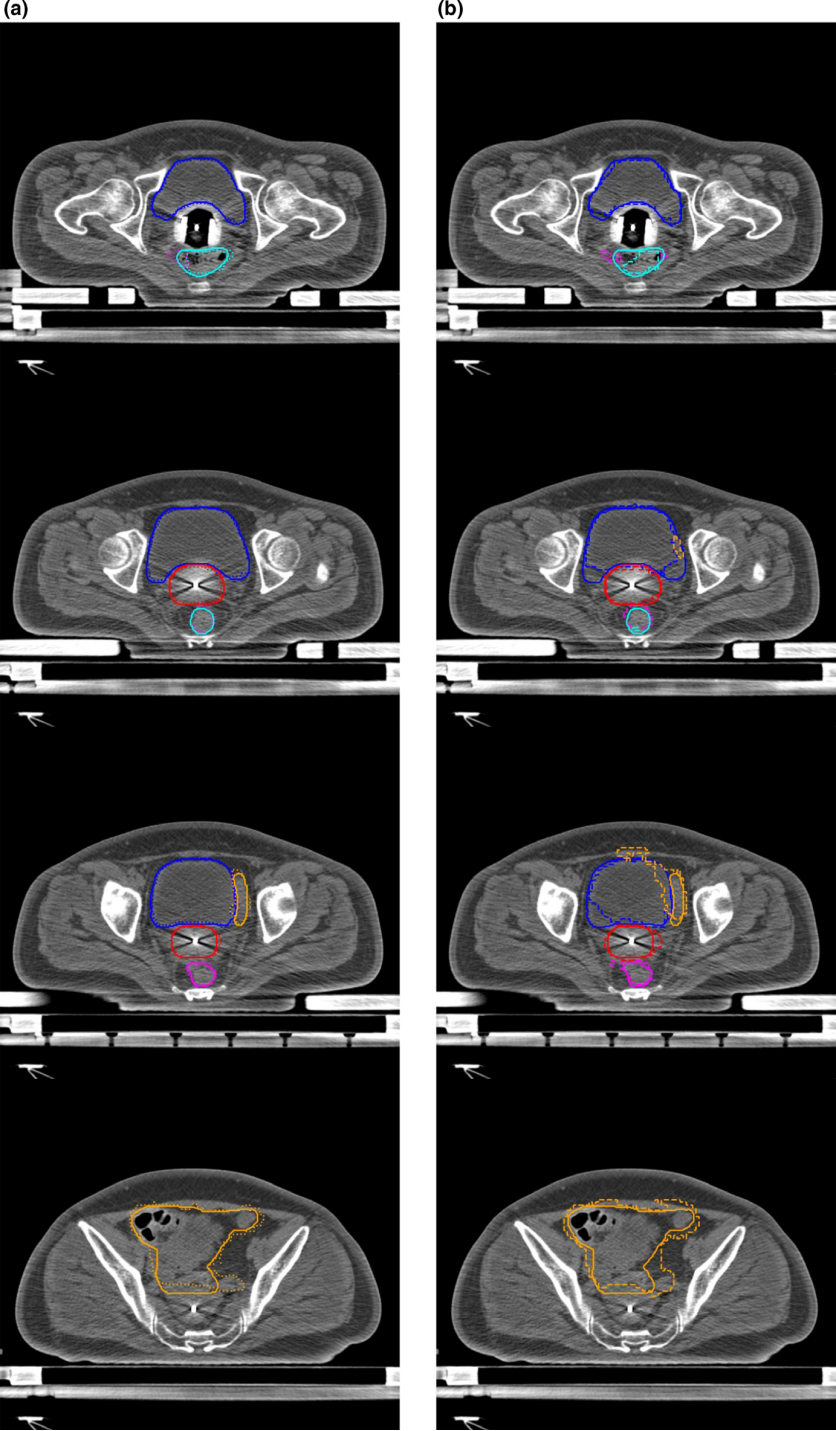
Segmentation results of a representative case (testing case 5). (a) shows the segmentation results with DSD‐UNET, (b) shows the segmentation results with 3D U‐Net. Solid lines indicate the ground‐truth segmentation, dotted and dashed lines indicate segmentations with DSD‐UNET and 3D U‐Net, respectively. (Blue contours: Bladder, Orange contours: Small intestine, Magenta contours: Sigmoid, Cyan contours: rectum, red contours: HR‐CTV)

**Fig. 4:**
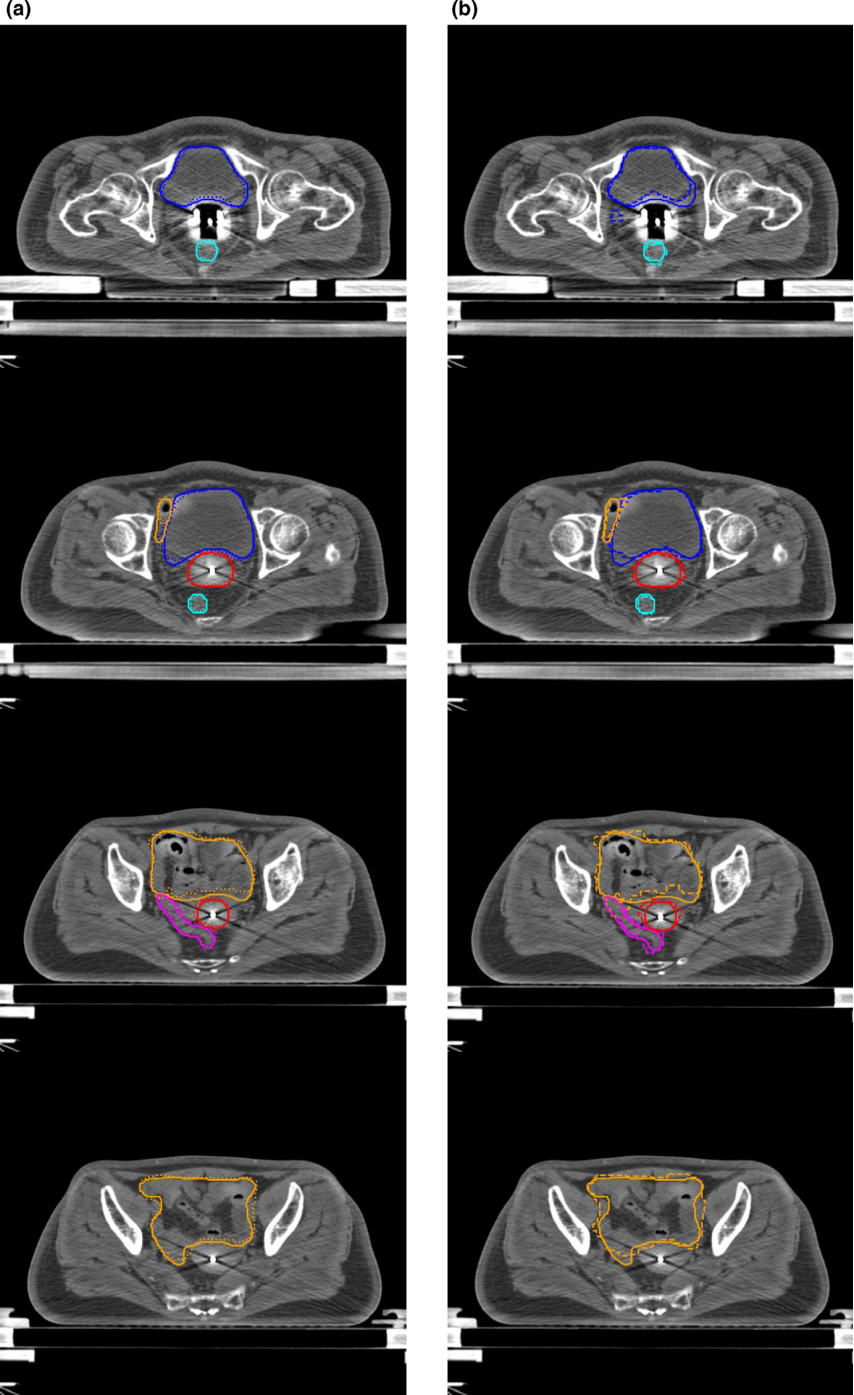
Segmentation results of a representative case (testing case 8). (a) shows the segmentation results with DSD‐UNET, (b) shows the segmentation results with 3D U‐Net. Solid lines indicate the ground‐truth segmentation, dotted and dashed lines indicate segmentations with DSD‐UNET and 3D U‐Net, respectively. (Blue contours: Bladder, Orange contours: Small intestine, Magenta contours: Sigmoid, Cyan contours: rectum, red contours: HR‐CTV)

**Fig. 5:**
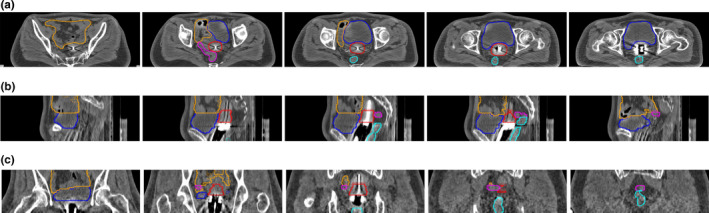
Segmentation results with DSD‐UNET model shown in transverse (a), sagittal (b) and coronal (b) views for a representative case (testing case 8). Solid lines indicate the ground‐truth and dotted lines indicate the prediction from DSD‐UNET. (Blue contours: Bladder, Orange contours: Small intestine, Magenta contours: Sigmoid, Cyan contours: rectum, red contours: HR‐CTV)

Table 1 shows the quantitative evaluations of segmentation results on the testing dataset with our network and 3D U‐Net. It can be found that the proposed DSD‐UNET model outperformed the 3D U‐Net model on segmentations of all the structures. The mean DSC values of the segmentation results with DSD‐UNET model were all bigger than 80.0% except for the sigmoid. The average DSC and JI values of DSD‐UNET were 7.0% and 9.2% higher than those of 3D U‐Net, respectively. The average HD value of DSD‐UNET was 3.7 mm lower than that of 3D U‐Net. Among all structures, the best results were obtained for bladder segmentation with mean DSC and JI values of 86.9% and 77.9%, respectively, for the DSD‐UNET model (DSC = 80.2% and JI = 68.2% for 3D U‐Net). This is mainly because the bladder has a relatively regular shape and clear boundary in the planning CT images. Automatic segmentation with DSD‐UNET also achieved a good result for HR‐CTV, the mean DSC and JI values reached 82.9% and 72.2%, respectively, the mean HD value was 8.1 mm. Segmentation of the rectum with DSD‐UNET model showed relatively good agreement with the GT segmentation, with mean DSC and JI values of 82.1% and 71.5%, respectively, and mean HD value of 9.2 mm. For the segmentation of the small intestine, mean DSC value of 80.3% was obtained with the DSD‐UNET model. However, the corresponding mean HD value reached 27.8 mm, which indicated the inferior segmentation accuracy. The most inferior segmentation accuracies were observed on the segmentations of sigmoid with both DSD‐UNET and 3D U‐Net models (DSC = 64.5%, JI = 52.2% and HD = 19.6 mm for DSD‐UNET, DSC = 55.2%, JI = 42.8% and HD = 23.4 mm for 3D U‐Net). Figures 3 and 4 show the visualization results of automatic segmentations with DSD‐UNET and 3D U‐Net for two representative cases. Compared with 3D U‐Net, the automatically segmented contours with DSD‐UNET showed better agreement with the GT contours in shape, volume, and location. For better visualization, Fig. 5 shows the automatic segmentation results with DSD‐UNET in the transverse, sagittal, and coronal views for a representative case. Although the training time for DSD‐UNET model was about 150 h, the time spent on segmentation of all the structures with the trained DSD‐UNET model was about 20 s per patient.

**Table 1:** Quantitative evaluations of the automatic segmentation results with DSD‐UNET and 3D U‐Net for the HR‐CTV and OARs (Mean ± Standard deviation)

Metrics	Models	Regions of interest				
		HR‐CTV	Bladder	Small intestine	Sigmoid	Rectum
DSC (%)	DSD‐UNET	82.9 ± 4.1	86.9 ± 3.2	80.3 ± 5.8	64.5 ± 7.9	82.1 ± 5.0
	3D U‐Net	74.2 ± 6.2	80.2 ± 4.1	75.1 ± 6.7	55.2 ± 8.6	77.1 ± 6.2
JI (%)	DSD‐UNET	72.2 ± 4.3	77.9 ± 3.5	69.4 ± 6.1	52.2 ± 8.3	71.5 ± 5.2
	3D U‐Net	60.2 ± 6.5	68.2 ± 4.6	61.9 ± 6.9	42.8 ± 8.8	64.3 ± 6.5
HD (mm)	DSD‐UNET	8.1 ± 2.3	12.1 ± 4.0	27.8 ± 10.8	19.6 ± 8.7	9.2 ± 4.6
	3D U‐Net	10.5 ± 2.9	16.3 ± 4.2	32.2 ± 11.9	23.4 ± 12.7	12.8 ± 6.0

### 3.B Performance of the proposed approach for automatic applicator reconstruction

**Fig. 6:**
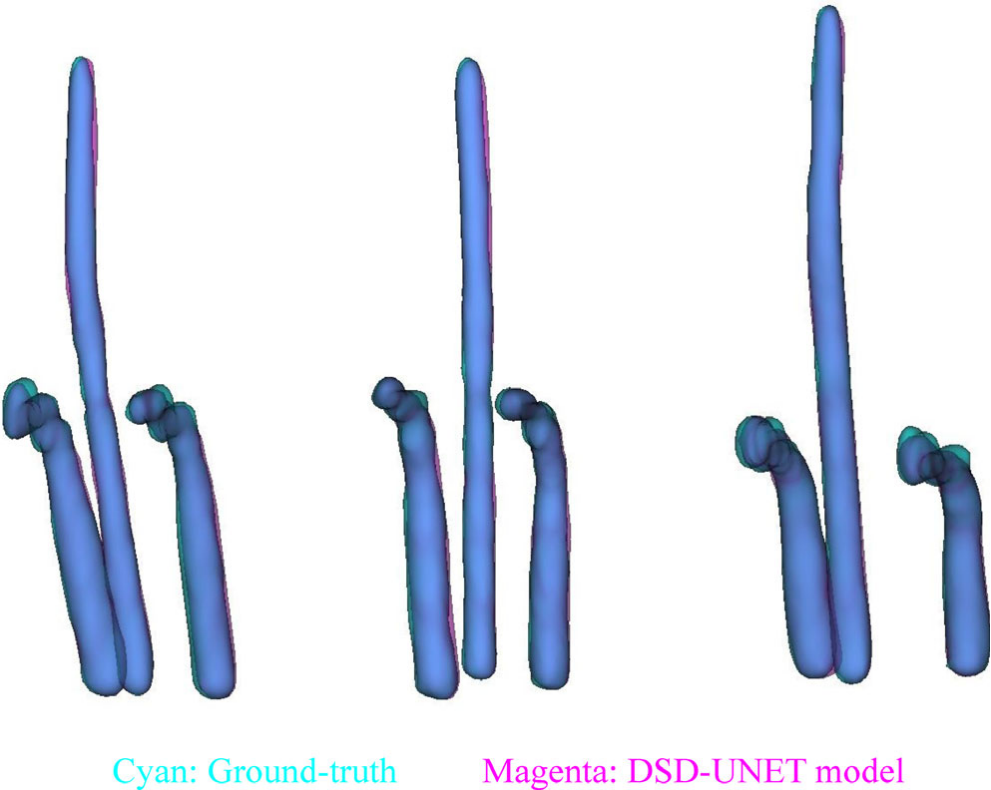
Three‐dimensional views of the segmentation results of intrauterine and ovoid tubes for three different cases. The ground‐truth and automatic segmentation results of the applicator tubes are displayed translucently in cyan and magenta, respectively, with the overlapped parts of the applicator tubes appearing blue

Segmentation results for all parts of the tandem and ovoid applicator using the DSD‐UNET model were assessed. The DSC, JI, and HD values computed on the testing set are reported in Table 2. It is observed that outstanding segmentation accuracies were achieved for all parts of the applicator. The mean DSC and JI values for all the applicator components were higher than 88.0% and 80.0%, respectively. In particular, automatic segmentations of the intrauterine tube and ovoid tubes achieved superior performances compared with those of the other applicator components (average DSC value of 92.1%, average JI value of 86.8%, average HD value of 2.3 mm). The best segmentation accuracy was observed on the segmentation of intrauterine tube (DSC = 92.6%, JI = 87.7% and HD = 1.9 mm). Three‐dimensional views of the segmentation results for intrauterine and ovoid tubes with the DSD‐UNET model are shown in Fig. 6. The segmentations of applicator tubes with DSD‐UNET model were in good agreement with the GT.

**Table 2:** Segmentation accuracy for all parts of the applicator with DSD‐UNET model (Mean ± Standard deviation)

	Intrauterine tube	Left ovoid tube	Right ovoid tube	Cervical stopper	Left ovoid	Right ovoid
DSC (%)	92.6 ± 1.3	91.9 ± 2.3	91.7 ± 1.8	88.3 ± 3.9	88.6 ± 4.1	88.9 ± 3.5
JI (%)	87.7 ± 1.4	86.8 ± 2.5	85.9 ± 2.1	80.1 ± 4.4	81.2 ± 4.2	81.5 ± 3.9
HD (mm)	1.9 ± 0.5	2.5 ± 0.8	2.4 ± 0.7	3.1 ± 1.6	3.2 ± 2.4	2.9 ± 2.6

**Fig. 7:**
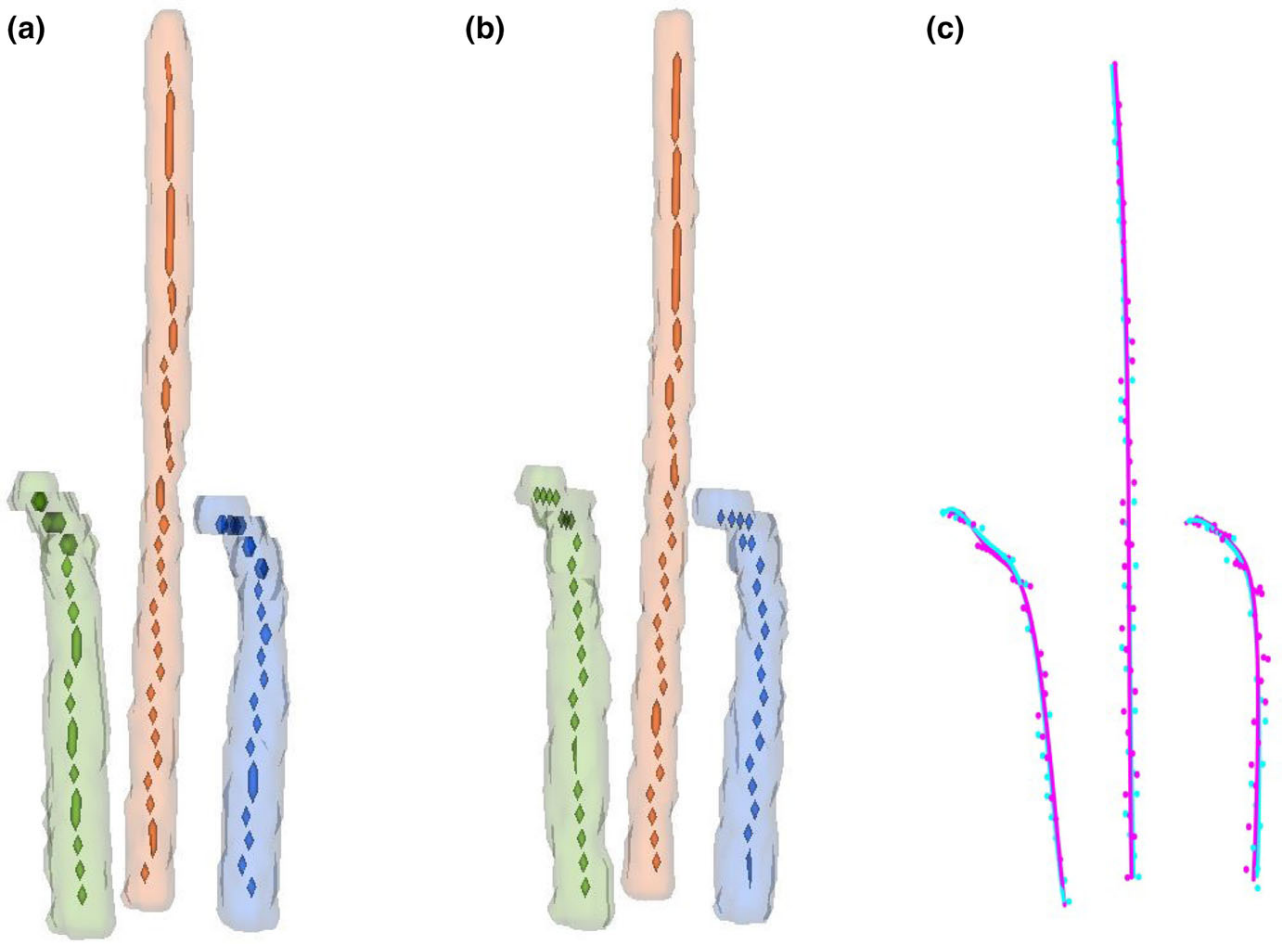
Illustration of the applicator reconstruction results for a representative case. (a) indicates the skeletonization of the applicator tubes for this case, the extracted skeletons are represented with voxel sets. (b) The manually determined points along the applicator channels for the same case. (c) Comparison of the polynomial curves fitted to the skeleton voxels (magenta) and manually determined points (cyan)

Following the automatic segmentation, skeletonization and polynomial curve fitting were conducted to obtain the channel paths of the applicator. HDs between the channel paths determined automatically and manually were 0.88 ± 0.12 mm (min‐max range 0.71–1.16 mm), 0.95 ± 0.16 mm (0.69–1.48 mm), and 0.96 ± 0.15 mm (0.75–1.34 mm) for the intrauterine, left ovoid, and right ovoid tube, respectively. The mean HD values for all the channel paths of the applicator were <1 mm, which indicated a good accuracy of the proposed method for automatic applicator reconstruction. Figure 7(a) illustrates the skeletons and segmentations of applicator tubes for an example case. The manually determined points along the applicator channels for the same case are shown in Fig. 7(b). The channel paths generated by the proposed method showed good agreement with the GT paths, as shown in Fig. 7(c). The computational time for the proposed automatic reconstruction approach was about 22 s per case.

## 4 DISCUSSION

Delineation of the target volumes and OARs is a critical step in radiotherapy treatment planning, which has important impact on not only the quality of treatment plan but also the clinical outcome. Therefore the fast and consistent auto‐segmentation method is highly desired and useful for treatment planning. Compared with automatic segmentation approaches based on multiple atlases, the CNN‐based automatic segmentation method is inherently a better solution that has strong generalization capability. Because it could capture multiple features and variations by itself, then build into the prediction model. In this study, we did not separate patients into different groups according to the size or shape of their body. The training and testing sets were chosen randomly. It was observed that relatively accurate and consistent segmentation results were obtained for all the testing cases. Computational results showed that the proposed DSD‐UNET method had strong ability of handling the input images with large differences. Compared with atlas‐based auto‐segmentation methods, another advantage of our method is higher segmentation efficiency. The DSD‐UNET architecture is an end‐to‐end segmentation framework which can directly provide voxel‐wise classification in CT images. The time for segmentation of all the structures with DSD‐UNET was about 20 s per patient. For atlas‐based auto‐segmentation methods, the indispensable processes of database search and deformable image registration are usually time‐consuming. Therefore, the proposed DSD‐UNET method is more efficient and clinically attractive.

In recent years, a variety of studies concerning automatic segmentation with CNNs in radiotherapy have been reported.^32^, ^33^, ^34^, ^35^, ^36^, ^42^ To the best of our knowledge, there were no reports on automatic segmentation for CT‐based BT of cervical cancer with any CNNs. This work is the first attempt to apply CNNs for this segmentation task. Moreover, automatic segmentation of HR‐CTV and OARs in the planning CT images for cervical cancer BT is more challenging for three main reasons. First, the metal artifacts caused by BT applicator degrade the image quality significantly. Second, the boundaries of HR‐CTV in the planning CT images for cervical cancer BT are hardly visible. Segmentation of the HR‐CTV depends largely on the physician’s knowledge. Third, the OARs in planning CT of cervical cancer BT (including bladder, small intestine, sigmoid, and rectum) show considerable changes in shape, volume, intensity, boundary, and location between patients. Nevertheless, relatively good segmentation results for the bladder and HR‐CTV were obtained with the proposed DSD‐UNET method, with the mean DSC values of 86.9% and 82.9% and the mean HD values of 12.1 and 8.1 mm, respectively.

We also compared the performance of DSD‐UNET model with that of the 3D U‐Net. Quantitative evaluations showed that the DSD‐UNET outperformed the 3D U‐Net in segmentation of all the structures. Superior performance of the proposed DSD‐UNET method was attributed to the novel architecture of the network and the additional input channel of preprocessed data. Specifically, a multipath dilated convolution module was deployed in the middle of the network to exploit the global context features that are essential for accurate segmentation. The dilated convolution module allowed the network to extract features in larger receptive fields without losing resolution. We also employed deep supervision in the expanding path of the network by integrating segmentation layers at different stages to form the final output. The combination of multilevel feature maps from shallow and deep layers of the network not only made the final segmentation more reliable but also accelerated the convergence of model training. In addition, activation function of LeakyReLU which allows a small and nonzero gradient when the unit is not active was used instead of conventional ReLU in our network. The introduction of LeakyReLU increased the expressiveness of DSD‐UNET network to some extent.

Segmentation result is closely related to the quality of input image for the automatic segmentation task. Therefore, the CLAHE algorithm was applied in this study to preprocess the input data for image enhancement. With the CLAHE method, the local contrast of original CT images increased significantly, the shape and boundary information of organs was highly improved without over‐amplification of noise. In order to preserve the original and essential information, we kept the original CT images with the enhanced images to compose a dual‐channel input to the network. The design of dual‐channel input facilitated the network to learn more substantial and valuable features from CT images.

In this study, the proposed DSD‐UNET architecture was also successfully exploited to segment all parts of the tandem and ovoid applicator in the planning CT images for cervical cancer BT. Accurate segmentation was achieved with the small training set of only 24 patients. It is demonstrated that the DSD‐UNET has strong ability in this segmentation task. Following the segmentation, channel paths of the applicator were obtained by the 3D skeletonization and polynomial curve fitting processing. To the best of our knowledge, it is the first attempt to employ CNNs to achieve automatic applicator reconstruction for cervical cancer BT. Different from the applicator model assisted reconstruction method that needs manual registration, the proposed method is fully automatic without any manual intervention. Therefore, more efficient and consistent applicator reconstruction can be achieved with our method.

For 3D image‐based BT, dose calculations are dependent on the geometrical accuracy of the source position relative to target volumes and OARs. The applicator displacements and reconstruction uncertainties could lead to major dose deviations in target and OARs due to the steep dose gradients of BT.^23^, ^24^, ^43^ It has been demonstrated that either ±3 mm displacement of the tandem and ovoid applicator or a ±4.5 mm applicator reconstruction uncertainty could cause greater than 10% dosimetry change for MRI‐based BT of cervical cancer.^25^ In order to minimize the reconstruction uncertainties and avoid accidental errors, implementation of automatic applicator reconstruction methods with high accuracy and consistency is necessary. In our study, automatic applicator reconstruction method utilizing the DSD‐UNET model has achieved relatively high accuracy. HD values between the channel paths determined automatically and manually were <1 mm for all the applicator channels.

In order to explore the feasibility and accuracy of the proposed method, we started the study with the relatively simple case of tandem and ovoid applicators. So the presented work serves as a preliminary exploration and simple test. Subsequent developments and more comprehensive evaluations are needed to extend the proposed method to more difficult scenarios.

One of the limitations of this work is the relatively small dataset size. This is due to the limited number of patients with cervical cancer that received CT‐based BT in our clinic and the lack of common dataset that is suitable for this segmentation task. To ease this problem, data augmentation strategy was applied in the model training. Dropout was deployed in the network to reduce the risk of overfitting introduced by data augmentation. However, due to the intrinsic characteristics of deep learning method, larger dataset usually leads to the improvements of performance and generalization. Therefore, we plan to collect more suitable image data in the future study. Then more accurate and reliable segmentation result could be expected.

The most inferior segmentation performance for the DSD‐UNET model was observed on the segmentation of sigmoid, with the lowest mean DSC value of 64.5% and higher mean HD value of 19.6 mm among all structures. With analysis of the segmentation results for sigmoid, we have found that misclassifications of the voxels from small intestine, sigmoid, and rectum were serious in most of the testing cases. The lower DSC value for segmentation of sigmoid is partly due to the misclassifications and the relatively small volume. It is really challenging to achieve accurate segmentation at the junction of small intestine and sigmoid as well as at the rectosigmoid junction by the proposed DSD‐UNET model. It is possible that the features extracted by DSD‐UNET are not sufficient to provide the desired segmentation result. Further investigation is needed to improve the accuracy of sigmoid segmentation.

For the automatic segmentation task, the original CT volume was cropped and resized to an input volume with fixed size of 128 × 128 × 64. This preprocessing was performed due to the limited computational resource and memory capacity available. However, the downsampling usually leads to a loss of detail information and may affect the segmentation accuracy. In the next stage of our work, we plan to feed the CNN with patches extracted from the original CT volume and stitch their respective outputs together to obtain the final segmentation. With this scheme, arbitrarily large images can be segmented without resampling and the training set can be enlarged to some extent. Moreover, we expect to train a single DSD‐UNET model that could segment both the anatomical structures and the applicator in the planning CT images, instead of two separated models in the current study. Therefore, the automatic segmentation and applicator reconstruction could be achieved simultaneously to streamline the treatment planning workflow for cervical cancer BT.

## 5 CONCLUSION

In this study, we presented a deep learning‐based method using DSD‐UNET architecture to automatically segment the HR‐CTV and OARs in the planning CT images for cervical cancer BT. Quantitative evaluation results show that the proposed DSD‐UNET method outperformed the 3D U‐Net and could segment the HR‐CTV, bladder, and rectum with relatively good accuracy. Moreover, the DSD‐UNET was exploited to segment the applicator components in the planning CT. Using 3D skeletonization and polynomial curve fitting, channel paths of the applicator were obtained without any manual intervention. The channel paths generated by our method show good agreement with the GT. HD between the channel paths determined automatically and manually was <1 mm. The proposed automatic segmentation and applicator reconstruction methods could be useful to improve the consistency and efficiency of treatment planning for cervical cancer BT.

## CONFLICT OF INTEREST

The authors have no conflict of interest to disclose.
